# Respiratory transmission potential of severe fever with thrombocytopenia syndrome bunyavirus: evidence from intranasal exposure in a humanized mouse model

**DOI:** 10.1080/22221751.2025.2511134

**Published:** 2025-05-23

**Authors:** Dafeng Lu, Yifang Han, Ruowei Xu, Chunfang Wang, Mingke Qin, Jianwei Shi, Fuqiang Ye, Jinhai Zhang, Zhenghan Luo, Yuhe Wang, Hong Lin, Peiqi Jia, Jin Zhu, Chunhui Wang

**Affiliations:** a School of Public Health, Capital Medical University, Beijing, People's Republic of China; b Department of infectious Disease Prevention and Control, Nanjing Bioengineering (Gene) Technology Centre for Medicine, Nanjing, People's Republic of China; c School of Life Sciences, Nanjing Normal University, Nanjing, People's Republic of China; d School of Public Health, Nanjing Medical University, Nanjing, People's Republic of China; e Department of Occupational Health, Third Military Medical University, Chongqing, People's Republic of China; f Department of Neurosurgery, Xuanwu Hospital Capital Medical University, Beijing, People's Republic of China; g Department of Blood Research Center, Jiangsu Province Blood Center, Nanjing, People's Republic of China; h Department of Clinical Laboratory, General Hospital of Eastern Theater Command, Nanjing, People's Republic of China

**Keywords:** SFTSV, humanized mouse model, respiratory transmission potential, intranasal drop challenge, next-generation sequencing

## Abstract

Severe Fever with Thrombocytopenia Syndrome Bunyavirus (SFTSV) is a highly lethal pathogen with expanding endemic regions in Asia. While primarily transmitted by ticks, recent evidence suggests potential airborne transmission, raising significant public health concerns. This study investigates the potential for respiratory transmission and pathogenesis using humanized NCG mice inoculated with SFTSV via subcutaneous injection challenge (SIC) or intranasal drop challenge (IDC). Both groups demonstrated rapid systemic dissemination, marked by viremia, weight loss, and multi-organ injury, with hemorrhagic manifestations observed in high-dose infection groups. Histopathological evaluations revealed lung pathology in the intranasal drop challenge mice, including extensive alveolar disruption and inflammatory cell infiltration. Transcriptomic analyses further confirmed that respiratory route inoculation resulted in heightened expression of inflammatory signalling pathways such as IL-17 and NF-κB, potentially contributing to severe local immunopathology. Subcutaneous infection provoked an earlier systemic immune response, with significant upregulation of antigen-processing genes in peripheral blood mononuclear cells. Nevertheless, both routes ultimately culminated in widespread injury to the liver, spleen, kidney, highlighting the systemic nature of SFTSV pathogenesis. These findings underscore the need for preventive strategies addressing respiratory spread.

## Background

Severe Fever with Thrombocytopenia Syndrome (SFTS) is a highly lethal infectious disease caused by Dabie bandavirus, initially discovered in the Dabie Mountains of China in 2009, also known as Severe Fever with Thrombocytopenia Syndrome Bunyavirus (SFTSV) [1–3]. Subsequent studies have unveiled varying levels of endemicity across distinct regions of China, signalling an expansion of affected areas and a rise in morbidity rates over recent years [2]. Reports have further emanated from other Asian territories, including Taiwan (China) [4], Japan [5]and South Korea [6]. Data reveal that the cumulative mortality rate of SFTS infections in China approximates 5.11% with 18,902 cases [6]. Other regions exhibit mortality rates ranging between 6.3% and 30% [2].

While ticks are the primary vectors for SFTSV transmission [1,7], epidemiological evidence indicates that human-to-human spread can occur through contact with blood, secretions, or excreta of infected patients [8–11]. Recent case reports highlight the possibility of airborne transmission or respiratory route transmissibility. Outbreak investigations in Xinyang (China) [12], South Korea [13], and Zhejiang (China) [14] suggest that SFTSV may infect individuals through droplets or mucosal membranes, even when standard protective measures are in place. Additionally, study has confirmed the airborne transmission potential of SFTSV through a mouse aerosol infection model [15]. These findings raise concerns that conventional tick-bite prevention alone may be insufficient to curb SFTSV spread.

Although observations suggest the possibility of aerosol transmission, limited research has been conducted on the capacity and mechanisms of SFTSV respiratory route transmissibility. To explore this, a nose-drop inoculation model was established to study SFTSV infection through the upper respiratory route mucosa or lungs. Traditional mouse models often fail to replicate human immune responses [15,16]. To address this limitation, humanized NCG mice were used [17]. These mice better simulate human immunity, providing a more relevant platform to study SFTSV infection [18,19]. The study compared infection outcomes between subcutaneous injection challenge (SIC) and intranasal drop challenge (IDC). Key parameters such as viral load dynamics, organ injuries, immune responses, and disease outcomes were analyzed to determine whether SFTSV can establish infection through the respiratory tract. The findings aim to improve understanding of SFTSV pathogenesis and guide public health strategies and therapeutic interventions.

## Method

### Viruses, cells, and animals

In this study, the SFTSV-JS-14 virus strain was obtained from the Jiangsu Provincial Center for Disease Control and Prevention. Vero cells (provided by the Naval Medical University) were cultured in DMEM (Gibco, Beijing, China) supplemented with 5% fetal bovine serum, 100 U/ml penicillin, and 100 μg/ml streptomycin. Employing gene editing techniques, NCG (NOD/ShiLtJGpt-*Prkdc*^em26Cd52^*Il2rg*^em26Cd22^/Gpt, GenPharmatech Co., Ltd, Nanjing, China) mice were engineered based on the NOD/ShiltJGpt strain, with knockouts of Prkdc (DNA-activated protein kinase) and Il2rg (common gamma-chain receptor) genes, resulting in low B cell and T cell levels [17,20].

### Establishment of humanized mouse infection model

Each mouse was administered an intraperitoneal injection of 500 μl of Peripheral Blood Mononuclear Cells (PBMCs) at a concentration of 3 × 10^7^/ml [17,21]. 100 μl blood sample was collected on day 21 for humanization assessment via flow cytometry. Humanization was confirmed when CD3 ^+^ CD45^+^ cells of PBMCs exceeded 8% [17].

The humanized mice were assigned to the following groups: SIC (5 × 10^5^ TCID_50_ SFTSV, n = 10), high-dose IDC (5 × 10^5^ TCID_50_, n = 10), moderate-dose IDC (5 × 10^4^ TCID_50_, n = 10), low-dose IDC (5 × 10^3^ TCID_50_, n = 10), SIC sequencing (5 × 10^5^ TCID_50_, n = 8), IDC sequencing (5 × 10^5^ TCID_50_, n = 8), and control (n = 12). IDC mice received 50 μl of SFTSV via nasal drops post-anesthesia, while SIC mice received 50 μl subcutaneously. All mice were housed in specific pathogen-free (SPF) facility at 22–24°C, with 50–70% relative humidity and a 12-hour light cycle (8:00–20:00). Their weight, temperature, activity, and food intake were recorded daily.

Non-sequencing group: On days 0, 1, 3, and 7 post infection (dpi), 150 μl of blood was collected from each mouse. The plasma collected on dpi 0 was previously used for flow cytometry. On dpi 14, surviving mice were euthanized, and blood was collected along with tissues (heart, lung, spleen, liver, kidney, testis, and brain). Any mouse that displayed severe adverse symptoms or died prematurely was immediately sampled by the same procedure. Sequencing group: No daily blood collection was performed. On dpi 3 and 7, three mice from each infected group were selected for blood collection and euthanasia, followed by tissue sampling. On dpi 3, three control mice were randomly chosen for the same procedure.

Blood samples were mixed with EDTA and stored at 4°C. Tissue samples were either stored in liquid nitrogen or fixed in paraformaldehyde for subsequent histological analysis. The detailed flowchart was provided in Appendix Figure 1. The flow cytometry results of humanization were shown in Appendix Figure 2.

### Enzyme-linked immunosorbent assay (ELISA) for plasma antibody and cytokine detection

Commercial ELISA kits were used to measure SFTSV-specific antibodies (Human SFTSV Ab Kit, JM-03042M2, Nanjing Jinmei, China), IL-6 (Human IL-6 ELISA Kit, LA137702H, Nanjing Lapuda, China) and Tumor necrosis factor α (TNF-α) (Human TNF-α ELISA Kit, LA137701H, Nanjing Lapuda, China) levels in plasma. Plasma was diluted due to limited volume. The minimum detection limits were 30 pg/ml for SFTSV antibody, 15.5 pg/ml for IL-6, and 117 pg/ml for TNF-α.

### Real-time polymerase chain reaction (PCR) for viral load detection

100μg Samples were finely pulverized and mixed with 900 μl of physiological saline, while plasma was diluted from 20 μl to 200 μl. SFTSV RNA from both tissue and plasma was extracted using the QIAamp MinElute Virus Spin Kit. The viral load in tissues and plasma was determined using the SFTSV RNA detection kit (Detection Kit for Severe Fever with Thrombocytopenia Syndrome Bunyavirus, DA0340, Daan Gene, China). The minimum detectable concentrations were 5 × 10^3^ copies/ml for plasma and 5 copies/mg for tissue.

### Hematoxylin and eosin (H&E) staining

Tissues (lung, liver, spleen, kidney, and brain) were fixed in paraformaldehyde for 24 hours, paraffin-embedded, sectioned, and stained with hematoxylin and eosin (H&E). For lung tissues, injury was semi-quantitatively scored (1–5 scale) based on alveolar integrity and inflammatory infiltration (including macrophages, lymphocytes, neutrophils, eosinophils, pleuritis, bronchiolar and perivascular inflammation, edema, and epithelial necrosis). The average scores were classified as mild (1, +), moderate (2–3, ++), or severe (4–5, +++), and pathological differences among IDC, SIC, and control groups were compared based on these scores. Histopathological changes including structural integrity, inflammatory cell infiltration, cellular necrosis, and morphological abnormalities were qualitatively assessed in liver, spleen, kidney, and brain tissues, and the differences among IDC, SIC, and control groups were comparatively analyzed.

### Next-generation sequencing

After euthanasia, RNA was extracted from PBMCs and lung tissues of the sequencing and control groups (RNeasy Plus Mini Kit, QIAGEN), with concentration evaluated by Qubit, purity and integrity by Agilent 5400. Library preparation and sequencing were performed on the Illumina NovaSeq 6000 platform. The data were aligned to a reference genome using HISAT2 (v2.0.5), and read counts per gene were calculated with featureCounts (1.5.0-p3). Differentially expressed genes were identified via edgeR (3.22.5) with a cutoff of |log2(FC)| ≥ 1 and padj ≤ 0.05. GO (Gene Ontology) and KEGG (Kyoto Encyclopedia of Genes and Genomes) pathway enrichment analysis was performed using clusterProfiler (3.8.1).

### Statistical analysis

All experimental data were analyzed using R software. Unless otherwise specified, *P* ≤ 0.05 was considered statistically significant.

### Laboratory safety and ethics

All experiments involving infected animals and handling of infectious samples were conducted in the BSL-3 laboratory, while nucleic acid testing was performed in the BSL-2 laboratory. The animal experiments were approved and registered by the relevant CDC facility, adhering to the ARRIVE guidelines and Directive 2010/63/EU of the European Parliament on the protection of animals used for scientific purposes.

## Results

### Rapid onset of infection symptoms post SFTSV intranasal exposure

**Figure 1.**
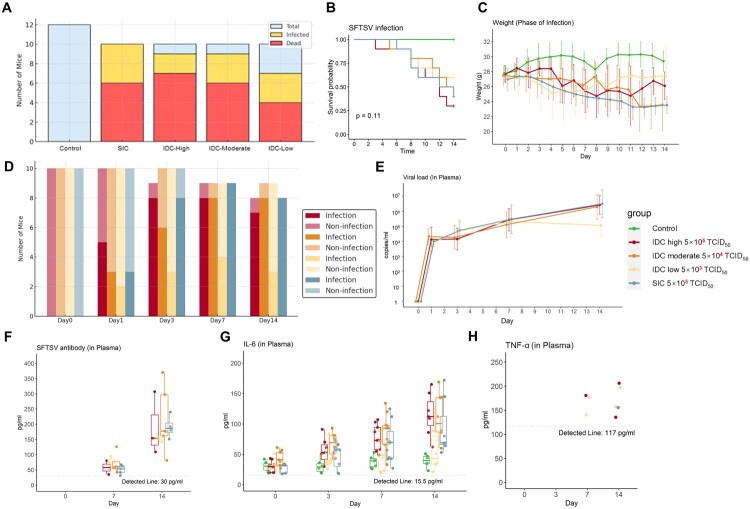
Progression of SFTSV Infection in Mice: Survival, Viral Load, and Immune Response Dynamics Across Infection Groups. A. Distribution of total, infected, and deceased mice across experimental groups. B. Survival probability following SFTSV infection across different infection routes and doses (log-rank test, *p* = 0.11). C. Longitudinal changes in body weight during infection. D. Infection status of individual mice over time by group. E. Plasma viral load (copies/mL) measured at multiple time points post-infection. F. SFTSV-specific antibody levels (pg/mL) in plasma at days 0, 7, and 14. G. Plasma IL-6 concentrations (pg/mL) in individual mice over time. H. Number of mice with elevated TNF-α levels above 117 pg/mL detection threshold. Groups: Control (*n* = 12), SIC (subcutaneous injection challenge group, *n* = 10), IDC-High (high dose intranasal drop challenge group: 5×10^5^ TCID₅₀, *n* = 10), IDC-Moderate (moderate dose: 5×10⁴ TCID₅₀, *n* = 10), IDC-Low (low dose: 5×10³ TCID₅₀, *n* = 10)

After SFTSV exposure, infection symptoms quickly appeared in mice. Both SIC and IDC routes induced infections. In the IDC-high group, 9 mice were infected (9 tested positive for viral RNA, 3 positive for SFTSV-specific antibodies), and 7 mice died during the observation period. In the IDC-moderate group, 9 mice were infected (8 tested positive for viral RNA, 5 positive for antibodies; notably, 1 mouse was RNA-negative but antibody-positive), and 6 mice died during the observation period. In the IDC-low group, 6 mice were infected (5 tested positive for viral RNA, 2 positive for antibodies; notably, 1 mouse was RNA-negative but antibody-positive), and 4 mice died during the observation period. In the SIC group, all 10 mice were infected (10 tested positive for viral RNA, 5 positive for antibodies), and 6 mice died during the observation period. (Figure 1A and B). Most deaths occurred in the second week (Figure 1B). Symptoms such as bloody stool, subcutaneous hemorrhage, hematuria, and cerebral hemorrhage were observed in 6 mice, including 3 in the high-dose IDC group, one in the moderate-dose IDC group, and 2 in the SIC group. (Appendix Figure 3) Additionally, weight loss was consistently observed throughout the infection period in the SIC and IDC groups, particularly in the high-dose IDC group and moderate-dose IDC group (*P* < 0.05) (Figure 1C). In the control group, no fatalities or symptoms were observed, and they showed a slight increase in weight (Figure 1A, B and C).

Assessments showed that on day 1 before exposure, no SFTSV RNA was detected in any of the mice. By dpi 1, infection was detected in each group, with positive rates ranging from 20% to 50%, and infection rates continued to rise over time. By dpi 14, detection rates in the infected groups ranged from 50% to 100% (Figure 1D). In all infected groups, plasma viral loads increased over time across all infection groups (*P* < 0.001) (Figure 1E), but with no significant differences between the groups (Appendix Figure 4A). SFTSV antibody detection showed no antibodies present on day 0. With disease progression, virus-specific antibody levels in infected mice notably increased. At dpi 7, antibody concentrations ranged from 30 to 100 pg/mL across infected groups, rising further to 100–370 pg/mL by dpi 14 (Figure 1F).

Plasma Interleukin 6 (IL-6) concentrations increased significantly across IDC high, IDC moderate and SIC groups as the infection progressed (Figure 1G and Appendix Figure 4B). Notably, in dpi14, IL-6 levels in the IDC high and IDC moderate groups remained significantly higher compared to the IDC low group (Appendix Figure 4C). TNF-α detection was sporadic, with one instance in the high-dose IDC group and two in the moderate-dose IDC group initially. By dpi 14, TNF-α was detected 2 in both the high-dose and moderate-dose groups, and 1 in the SIC group (Figure 1H).

### SFTSV invaded multiple organs and caused substantial injury

**Figure 2.**
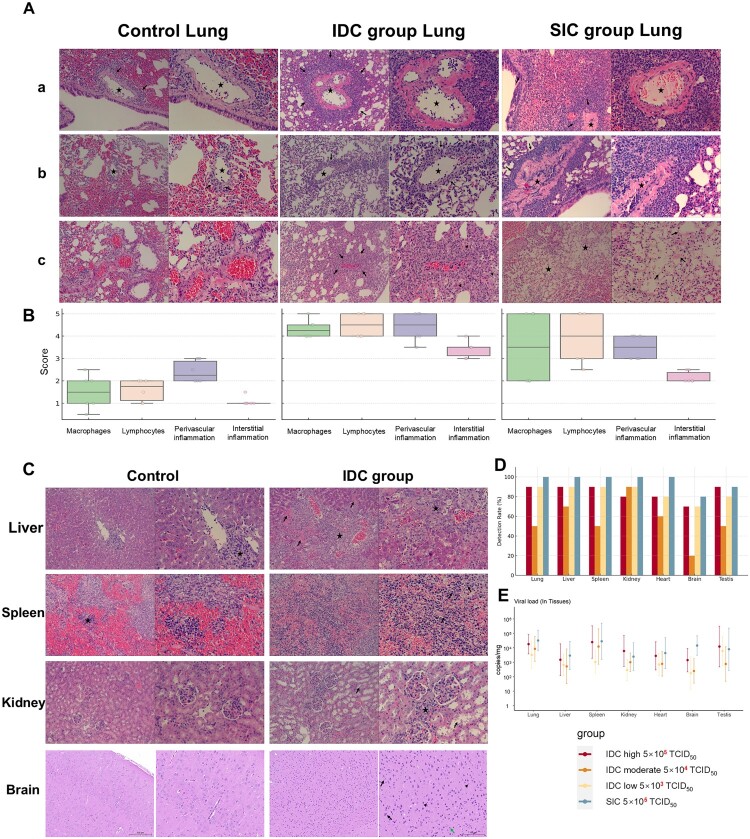
Histopathology and Viral Dynamics in Tissues of SFTSV-Infected Mice. A: Comparative Histopathology of Lung Tissues Control Group: Lung Tissue: Lung tissue exhibits intact pulmonary architecture, well-defined bronchioles, tightly arranged blood vessels, and clearly structured alveoli. Alveolar spaces show moderate and uniform expansion, with mild perivascular inflammatory cell infiltration (long arrows) observed. Lung IDC Group: a: Lung sections display moderate histopathological changes, characterized by focal mononuclear cell infiltration (long arrows) extending into alveolar and perivascular regions. b: Severe structural disruptions in alveolar integrity are observed, accompanied by fibrin-like exudates and necrotic cellular debris. c: Numerous infiltrating cells with eosinophilic cytoplasm (long arrows) and abundant foamy cells (arrowheads) are observed. The overall pulmonary structure shows severe damage indicative of exacerbated viral pathogenicity. Lung SIC Group: a and b: Lung tissues demonstrate severe pathological alterations, including extensive mononuclear cell infiltration (long arrows), pronounced alveolar structural disruption, and indistinct vascular boundaries. c: Multiple focal infarctions (pentagrams) occur within alveolar regions, characterized by extensive epithelial and endothelial cell necrosis, fibrin-like remnants (long arrows), and prominent inflammatory exudates. B: Inflammatory Scores of Lung Tissue Lung injury was evaluated using a semi-quantitative scoring system (1-5) based on key histopathological features. Shown are the average scores for four representative indicators: macrophage infiltration, lymphocyte infiltration, perivascular inflammation, and interstitial inflammation, across control, SIC, and IDC groups. The boxplots reveal distinct inflammatory patterns among the groups, with IDC lungs exhibiting the most pronounced changes. C: Comparative Histopathology of Liver, Kidney, Spleen, and Brain Control Group: Liver Tissue: Neat hepatocyte arrangements with no abnormalities. Mild focal infiltration of mononuclear cells is seen around the central vein, with clustered distribution (pentagrams). Spleen Tissue: Clearly defined red and white pulp regions, with a normal lymphocyte count. Occasional clusters of extramedullary hematopoietic cells are observed in the red pulp (pentagrams). Kidney Tissue: Orderly structures, with well-organized renal tubules and glomeruli. Brain Tissue: Healthy neuronal integrity, with normal neuron size, Nissl bodies, and moderate glial cells. Infection Group: Liver Tissue: Pericentral hepatocyte necrosis with inflammatory cell infiltration (pentagrams). Histological structure is disrupted, with hepatic cords replaced by inflammatory cells. Necrotic hepatocytes display strong eosinophilia with nuclear pyknosis, karyorrhexis, or karyolysis (long arrows). Spleen Tissue: Loosely organized structure; blurred red and white pulp boundaries; shrunken lymphocytes detached from tissues (long arrows). Kidney Tissue: Dilated renal tubules (long arrows); mononuclear cell clusters with eosinophilic cytoplasm around glomeruli (pentagrams). Brain Tissue: Disorganized structure; reduced neurons with eosinophilic cytoplasm, Nissl body loss, satellite cells (long arrows), activated microglia (arrowheads), and neutrophils (green long arrows). D: Tissue-Specific Detection Rates of SFTSV in Infected Mice. E: Tissue Viral Loads Across Experimental Groups.

Histopathological examination revealed substantial organ damage induced by SFTSV infection, with lung tissues showing clear differences across groups (Figure 2A). Control group lungs showed intact alveolar structures with mild inflammation. SIC group lungs exhibited increased infiltration of macrophages and lymphocytes, predominantly moderate-to-severe, along with notable perivascular inflammation and moderate interstitial inflammation. IDC group lungs presented the highest severity, characterized by extensive inflammatory infiltration predominantly scored as severe, widespread perivascular inflammation, and marked interstitial inflammatory responses (Figure 2B).

In the liver, there was noticeable hepatocyte necrosis around hepatic venules, along with massive macrophage infiltration replacing the hepatic cords. The spleen’s architecture was loosened, with a blurred demarcation between red and white pulp, and reduced lymphocyte volume with detachment from surrounding tissue. In the kidneys, mononuclear cell infiltration around the glomeruli disrupted the normal structure, while in the brain, neuronal details are obscured, and Nissl bodies are absent. Additionally, there was increased activation of microglial cells and neutrophil infiltration (Figure 2C). No substantial histopathological differences between IDC and SIC were observed for these tissues.

Tissue viral RNA detection results are shown in Figure 2D, which presents the detection rates across different groups. Viral load analysis indicated that the spleen showed the highest levels of virus, while brain tissues showed relatively lower loads. The highest viral burdens were found in the high-dose IDC and SIC groups (Figure 2E).

### Transcriptomic profile of mice infected via different routes

In this study, we performed transcriptomic sequencing of lung tissues and PBMCs from SIC and IDC groups at dpi3 and dpi7 aiming to compare the global and dynamic gene expression patterns induced by different infection routes. At dpi3, the IDC group showed 3684 differentially expressed genes (DEGs) in lung tissue (2017 upregulated, 1667 downregulated), while the SIC group had 3102 DEGs (1872 upregulated, 1230 downregulated). In PBMCs, 336 DEGs (115 upregulated, 221 downregulated) were found in the IDC group, versus 373 DEGs (171 upregulated, 202 downregulated) in SIC. By dpi7, DEG counts rose notably in the IDC lungs (4792 total; 2443 upregulated, 2349 downregulated) compared to SIC lungs (2919 total; 1533 upregulated, 1386 downregulated). PBMCs also showed increased DEGs in IDC (713 total; 290 upregulated, 423 downregulated) relative to SIC (304 total; 129 upregulated, 175 downregulated) at day 7. Volcano plots were provided in Appendix Figure 5 and 6.

**Figure 3.**
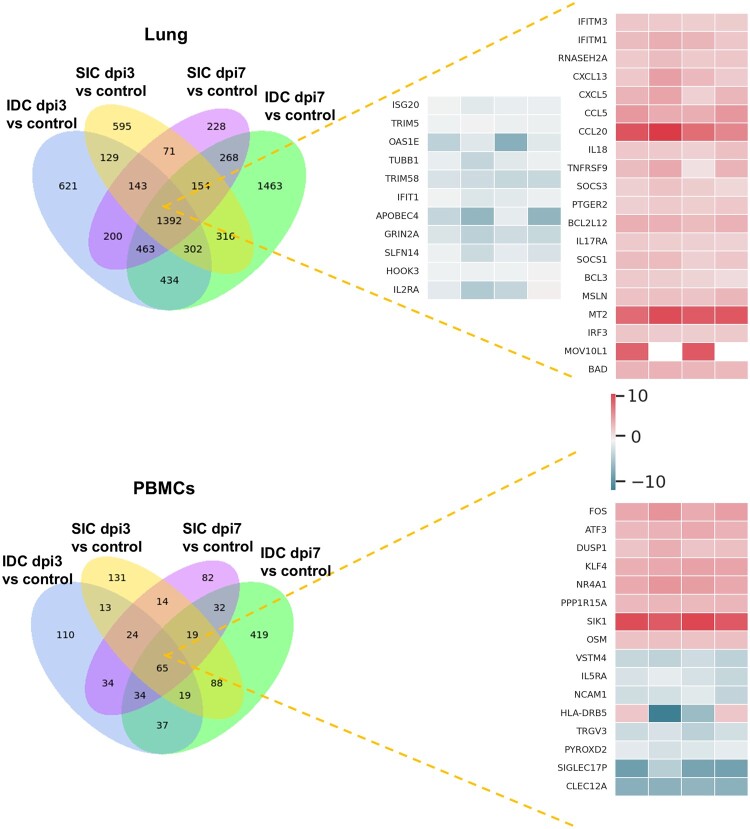
Overlap and Expression Patterns of Differentially Expressed Genes (DEGs) in Lung Tissues and PBMCs During SFTSV Infection. Venn diagrams (left) show the number of differentially expressed genes (DEGs) identified in the lung tissues and PBMCs at dpi3 and dpi7, across subcutaneous injection challenge (SIC) and intranasal drop challenge (IDC) routes compared to uninfected controls. Heatmaps (right) display representative DEGs from the overlapping regions, reflecting transcriptional responses in both compartments.

In lung tissue, several DEGs were consistently upregulated across both IDC and SIC groups, including genes such as *IFITM3*, *CXCL13*, *SOCS3*, and *MT2*. These genes are primarily involved in antiviral responses, chemokine signalling, and immune cell recruitment. Genes consistently downregulated (e.g. *OAS1E, TRIM5, IFIT1, ISG20*, etc.), associated with antiviral defense, immune regulation, and cellular structural integrity. In PBMCs, the consistently upregulated genes included *FOS, ATF3, KLF4, SIK1* and *NR4A1*, primarily related to inflammation regulation and transcription factor activation. Several genes, such as *VSTM4*, *CLEC12A*, *SIGLEC17P*, and *TRGV3*, were consistently downregulate. The Venn diagram and heatmap of DEGs are shown in Figure 3.

**Figure 4.**
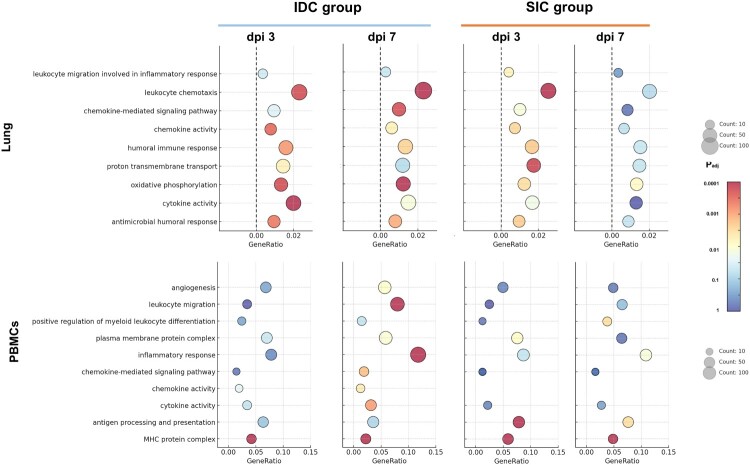
GO Enrichment Analysis of Differentially Expressed Genes in Lung Tissues and PBMCs at dpi3 and dpi7. Gene Ontology (GO) enrichment analysis was performed on differentially expressed genes (DEGs) identified in lung tissues (top panels) and PBMCs (bottom panels) at dpi3 and dpi7, in both subcutaneous injection challenge (SIC) and intranasal drop challenge (IDC). Dot plots display significantly enriched GO biological processes and molecular functions, with dot size representing the number of enriched genes and color indicating the adjusted p-value.

Gene Ontology (GO) enrichment analyses highlighted both common and distinct biological pathways activated by different infection routes and over the course of infection. In lung tissue, Several GO terms were enriched across both infection routes, including “antimicrobial humoral response”, “human immune response”, “leukocyte chemotaxis”, “cytokine activity” and “oxidative phosphorylation” indicating a general activation of innate immunity, inflammatory signalling, and metabolic adaptation during infection. However, distinct differences emerged between IDC and SIC infections. The IDC group exhibited marked enrichment of “antimicrobial humoral response”, “chemokine activity” and “oxidative phosphorylation”, suggesting that infection via the respiratory route induces a more pronounced stimulation in the lungs (Figure 4). In PBMCs, The MHC protein complex, antigen processing and presentation and plasma membrane protein complex pathways were commonly enriched across all groups, reflecting the activation of immune recognition, and intercellular signalling processes., suggesting that infection via the subcutaneous route may elicit a more direct stimulation of the systemic immune response. Compared with the SIC group, pathways related to chemokine activity and cytokine activity were more significantly enriched in the IDC group. Although the antigen processing and presentation pathway was enriched under both infection routes, its activation was relatively less prominent in the IDC group. Similarly, pathways associated with leukocyte migration and inflammatory response were enriched during the dpi7 stage. The enrichment was more pronounced in the IDC group (Figure 4).

**Figure 5.**
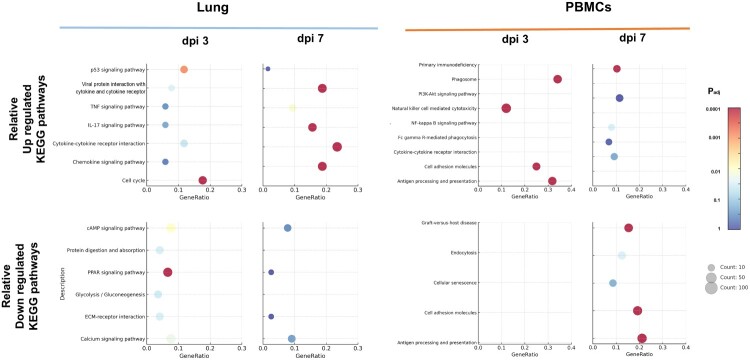
Comparative KEGG Pathway Enrichment Analysis Between IDC and SIC Groups in Lung Tissues and PBMCs. KEGG pathway enrichment analysis was performed to compare the transcriptomic differences between the subcutaneous injection challenge (SIC) and intranasal drop challenge (IDC) in both lung tissues (left) and PBMCs (right) at dpi3 and dpi7. Enriched pathways were separated into relatively upregulated (top) and downregulated (bottom) in the IDC group compared to the SIC group. Dot size represents the number of enriched genes per pathway, while color indicates the adjusted p-value.

Differential gene expression analysis between the IDC and SIC groups was conducted, followed by KEGG pathway enrichment analysis to explore their functional differences. In lung tissues (Figure 5), multiple inflammation and immunity related signalling pathways were significantly upregulated at dpi 3, including cytokine-cytokine receptor interaction, IL-17 signalling pathway, and pathways associated with viral protein – cytokine receptor interactions. In addition, pathways related to cell proliferation and homeostasis maintenance, such as the p53 signalling pathway and cell cycle, also showed an upward trend. By dpi 7, enrichment of the TNF signalling pathway and chemokine signalling pathway was observed, with further enhanced enrichment of the IL-17 signalling pathway, cytokine-cytokine receptor interaction and viral protein – cytokine receptor interaction pathway. In contrast, several pathways associated with energy metabolism and cellular signal regulation were relatively downregulated, including glycolysis/gluconeogenesis, ECM-receptor interaction, protein digestion and absorption, calcium signalling pathway, cAMP signalling pathway, and the PPAR signalling pathway in dpi3. Notably, protein digestion and absorption and calcium signalling pathway remained downregulated on dpi 7. In PBMCs (Figure 5), several immune-related pathways were enriched at dpi 3, including natural killer (NK) cell mediated cytotoxicity, cell adhesion molecules, phagosome, and antigen processing and presentation. These findings indicated that, during the early stage of infection, circulating immune cells exhibited a robust activation of recognition and cytotoxic responses against viral or pathogenic stimuli, along with enhanced cellular interactions, phagocytic activity, and antigen presentation functions. In the dpi 7, continued upregulation of immune regulatory pathways was observed in the IDC group, including primary immunodeficiency, PI3K-Akt signalling pathway, NF-κB signalling pathway, Fc gamma R-mediated phagocytosis, and cytokine-cytokine receptor interaction. In contrast, pathways related to cellular senescence, endocytosis, graft-versus-host disease, cell adhesion, and antigen presentation were relatively downregulated.

## Discussion

In this study, we aimed to compare the pathogenic and immunological consequences of SFTSV infection via intranasal drop and subcutaneous injection in a humanized NCG mouse model. Our comprehensive analysis integrates clinical signs, histopathological damage, plasma cytokine profiles, viral loads. High-throughput RNA-sequencing was performed on lung tissues and PBMCs from infected mice at dpi 3 and dpi 7. Our findings demonstrate that SFTSV, regardless of whether it enters via nasal mucosa or subcutaneous tissue, can rapidly proliferate in the host, leading to viremia and uncontrollable inflammation and hemorrhage. By dpi 14, plasma viral loads steadily increased in all groups, correlating with severe clinical manifestations. Hemorrhagic symptoms were common in both IDC and SIC infection groups, especially at moderate and high doses, suggesting a synergistic role of pulmonary inflammation in exacerbating systemic disease. Meanwhile, in the IDC group, the administered doses showed a significant correlation with infection rates, suggesting a link between SFTSV transmissibility and viral load.

Significant symptoms appeared swiftly after infection, that both IDC and SIC routes led to rapid onset of symptoms, with detectable viral RNA in peripheral blood by day dpi 1. Notably, the IDC route, despite its mucosal entry point, did not delay systemic dissemination, suggesting that SFTSV efficiently breaches epithelial barriers. Previous studies suggested that SFTSV primarily targets monocytes and dendritic cells (DCs) [22–24], and dendritic cell abundant in mucosal and lymphoid tissues [25,26]. This highlights a potential mechanism whereby SFTSV exploits DCs for respiratory route transmissibility, as DCs may inadvertently assist the virus in crossing epithelial barriers and entering the lymphatic system [10]. Once inside, SFTSV leverages the immune system’s cellular machinery for systemic dissemination, a phenomenon reminiscent of other viral infections such as Dengue and measles [27–29]. Nevertheless, further experimental evidence is needed to fully validate this proposed mode of transmission.

Histological examination revealed severe pulmonary injury in the IDC group, which exhibited more extensive mononuclear infiltration, alveolar wall thickening, and interstitial edema compared to the SIC group. This difference is plausible, as direct nasal inoculation presumably primes an immediate local immune response in lung, leading to localized immunopathology. These findings are further supported by transcriptomic data showing significant upregulation of proinflammatory pathways, including IL-17, TNF, and NF-κB signalling, in IDC lung tissues. IL-17, a key cytokine in mucosal immunity that promotes neutrophil recruitment and sustained inflammation [30–32], was notably elevated in IDC lungs, suggesting that respiratory infection uniquely triggers local cytokine storms contributing to alveolar damage and vascular leakage [33]. Notably, such localized inflammatory responses may drive systemic disease through increased vascular permeability and cytokine spillover into circulation [34,35]. In contrast, SIC lungs showed moderate inflammatory responses, with less severe histological disruption and relatively lower expression of inflammatory genes. By dpi 7, the synergy among IL-17, TNF, and NF-κB can set off a “cytokine storm,” fuelling alveolar necrosis, perivascular inflammation [36], and foam cell infiltration – pathological features consistent with those observed in the IDC group [33,37].

Beyond the lungs, SFTSV induced widespread histopathological damage across multiple organs, including the liver, spleen, kidneys, and brain. No substantial histopathological differences between IDC and SIC were observed for these tissues, likely because sampling predominantly occurred at advanced disease stages. Nonetheless, these tissues showed common pathological features, including hepatocyte necrosis, glomerular infiltration, and neuronal damage. In the spleen, blurred boundaries between the white and red pulp alongside significant lymphocyte depletion indicated a severe compromise of this critical immune hub, closely associated with the targeting of macrophages by SFTSV [38,39]. These macrophages, once heavily infected, not only failed to perform normal antigen presentation and pathogen clearance but also became replication sites for SFTSV [40,41], leading to extensive viral proliferation and systemic immunosuppression [34,42,43]. Moreover, immunopathological observations revealed that SFTSV infects neurons, inducing cerebral hemorrhagic lesions. Such findings align with neurological manifestations reported in SFTSV encephalitis cases [31] and mirror patterns observed in other viral brain infections, including COVID-19 [35].

Significant increases in plasma IL-6 levels during infection reflect a robust innate immune reaction to viral intrusion, consistent with previous findings [17,30]. Although TNF-α detection was suboptimal, it remained detectable in moderate – and high-dose groups at later stages; together, these cytokines promote vascular leakage and tissue necrosis, resulting in widespread hemorrhage [39,44–46].

Focusing on PBMCs, we observed that subcutaneous infection was strongly associated with the enrichment of “MHC protein complex” and “antigen processing and presentation” pathways [3,30], suggesting a more direct and earlier onset of systemic-specific immunity compared to IDC. Thus, while both routes effectively initiate immune activation, the SIC route appears to prime PBMCs earlier for systemic-specific responses, whereas IDC elicits a delayed but highly localized inflammatory storm in the respiratory tract.

The p53 signalling pathway and cell cycle processes were also impacted. While some upregulation of p53 was initially noted, the net effect over time seemed to be insufficient to curb viral replication. Proteomic studies [47] further highlight the upregulation of antiapoptotic proteins, such as SOD2 and BCL3, which suppress apoptosis and extend the survival of infected cells, creating a favourable environment for viral replication [30]. Moreover, the viral NSs protein plays a critical role in manipulating the host cell cycle, inducing G2/M phase arrest to optimize conditions for viral replication [38,48]. These combined strategies amplify tissue damage and systemic inflammation, contributing to the severe clinical manifestations of SFTSV infection, including multi-organ dysfunction and hemorrhagic symptoms.

Hemorrhage and organ injury highlight the challenges of treating severe SFTSV infection, emphasizing the importance of individualized therapeutic approaches. Suppressing hyperactive inflammatory pathways, such as IL-17 and NF-κB, should be balanced to avoid compromising antiviral defense. In parallel, targeting virus-induced suppression of apoptosis and cell cycle checkpoints, such as restoring p53 signalling, could enhance immune cell viability and regenerative capacity [49]. These strategies must be tailored to disease progression, ensuring early intervention focuses on viral load reduction, while later stages address immune exhaustion and tissue repair [50].

The study has several limitations. First, we used humanized NCG mice, which, while partially reflecting the human immune system, still have key immunological and physiological differences from humans, including a low B-cell level [51]. Second, due to sample volume and technical constraints, our plasma cytokine analysis focused on IL-6 and TNF-α, leaving out other important markers such as IL-10 and TGF-β. Third, although transcriptomic analyses indicated changes in immune-related pathways, we did not perform functional validation, so the exact roles of these pathways remain unclear. Finally, our study employed intranasal drop challenge (IDC) rather than direct animal-to-animal transmission experiments, which does not fully replicate the natural process of respiratory transmission, including aerosol generation, droplet spread, and subsequent infection.

In conclusion, this study employed a humanized NCG mouse model to investigate the pathogenic features and immunological responses of SFTSV via two routes of infection: subcutaneous injection challenge route or intranasal drop challenge route. The results indicate that regardless of whether the virus enters through intranasal or subcutaneous pathways, it rapidly disseminates systemically and induces severe immunopathological damage. The subcutaneous route triggered a more direct and earlier systemic immune response. The intranasal infection group displayed a more intense localized immune reaction in the lungs. Moreover, in both infection groups, multi-organ damage and hemorrhagic manifestations were observed at later stages. Transcriptomic data further revealed that intranasal infection prominently activates IL-17, TNF, and NF-κB, contributing to a more pronounced “cytokine storm” and tissue injury. By contrast, subcutaneous infection more strongly invoked antigen presentation and adaptive immune pathways. These findings confirm that SFTSV can efficiently utilize the respiratory route for transmission and cause severe pulmonary injury, highlighting a need for enhanced public health measures targeting both tick-bite prevention and respiratory protection.

## Supplementary Material

Appendix Figure.pdf

## Data Availability

The data generated or analyzed during this study are encompassed within this published article and its supplementary information files.

## Data sharing

Requests for extracted data can be directed to the corresponding author.
