# Supplementary material for: Respiratory transmission potential of severe fever with thrombocytopenia syndrome bunyavirus: evidence from intranasal exposure in a humanized mouse model
Source: Emerg Microbes Infect. 2025 May 23;14(1):2511134. doi: 10.1080/22221751.2025.2511134 (PMC12180321; doi:10.1080/22221751.2025.2511134)

Appendix Figure 1

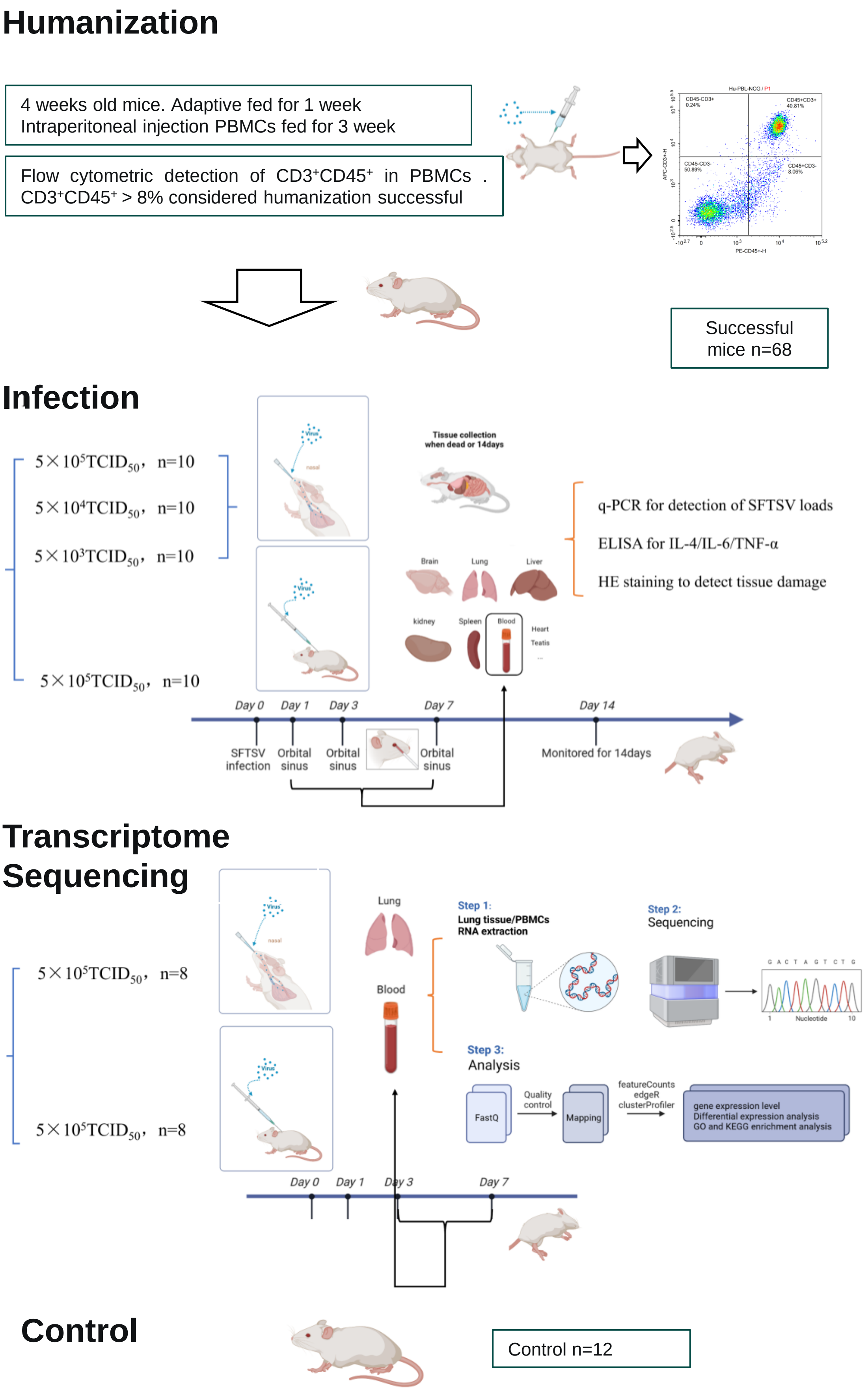

Appendix Figure 2

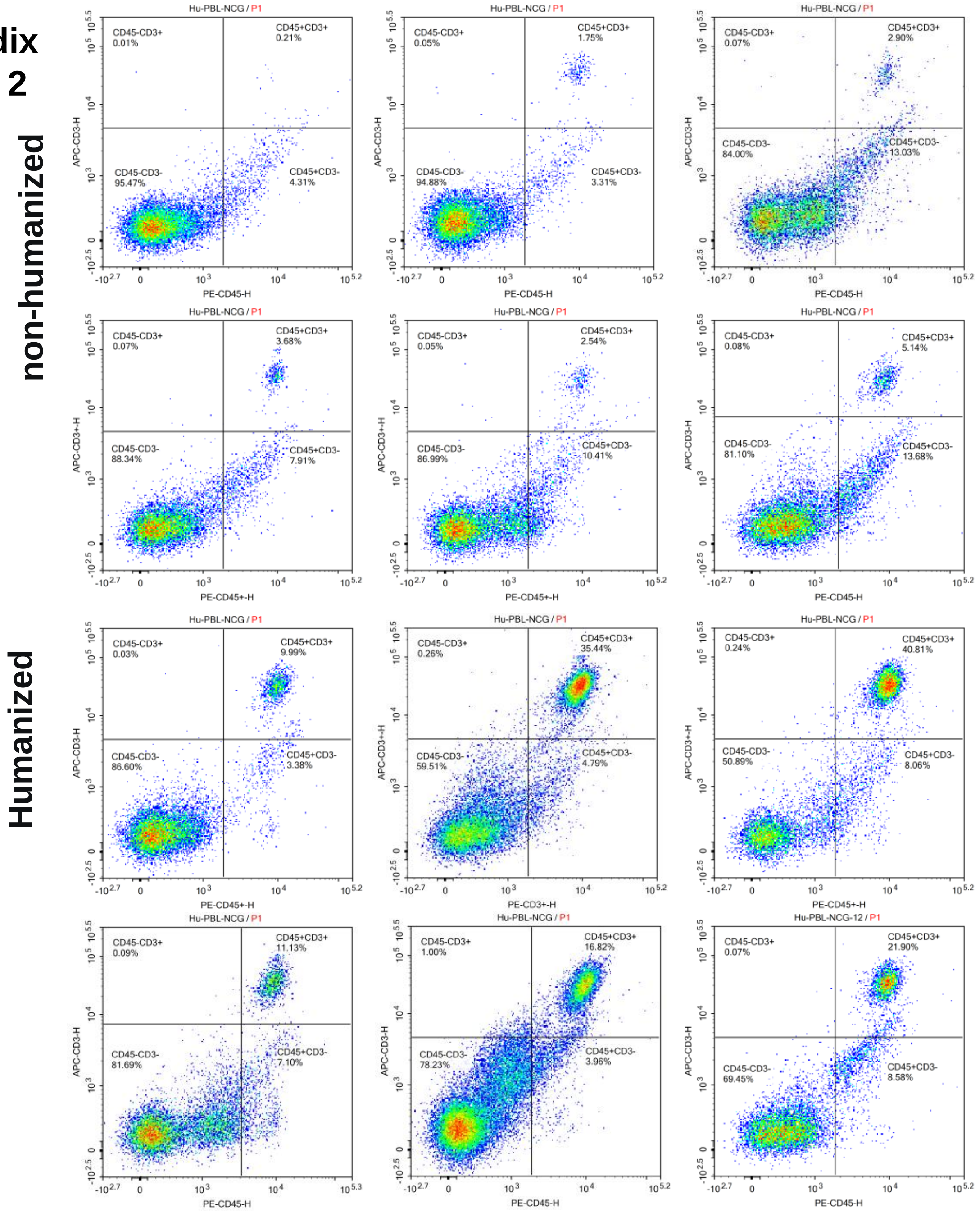

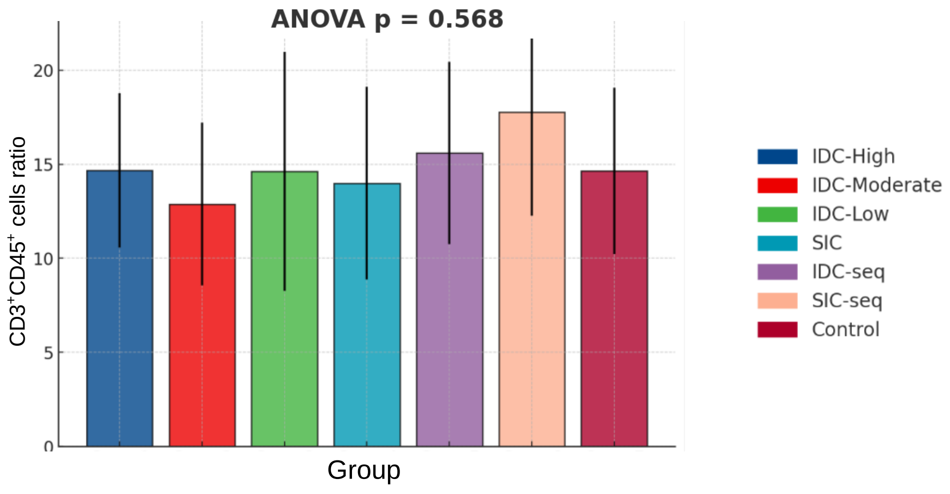

Appendix Figure 3

A

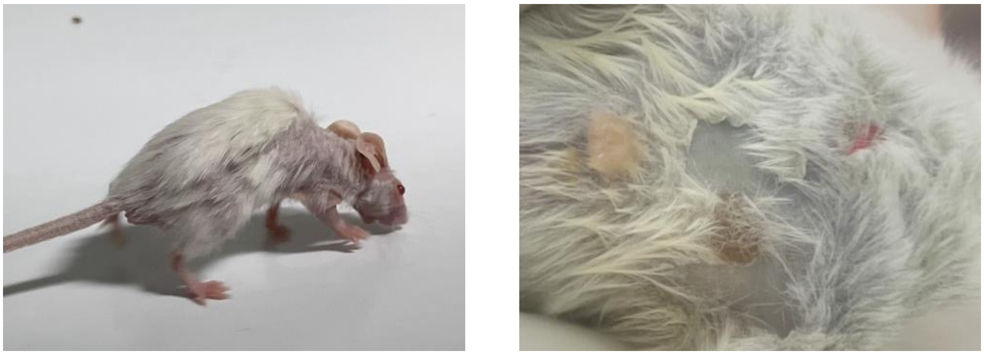

B

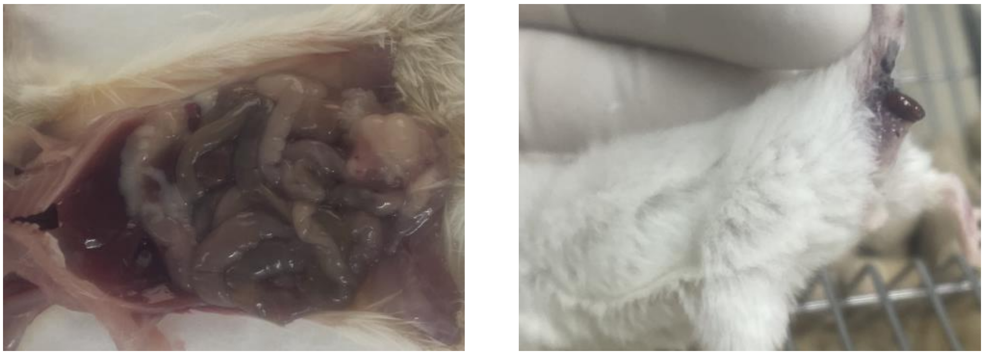

A After infection, some mice developed hair loss and skin lesions, with more severe manifestations observed in those that eventually succumbed.

B Bloody stool, subcutaneous hemorrhage, hematuria, and cerebral hemorrhage were observed in 6 mice, including 3 in the high-dose IDC group, one in the moderate-dose IDC group, and 2 in the SIC group.

Appendix Figure 4

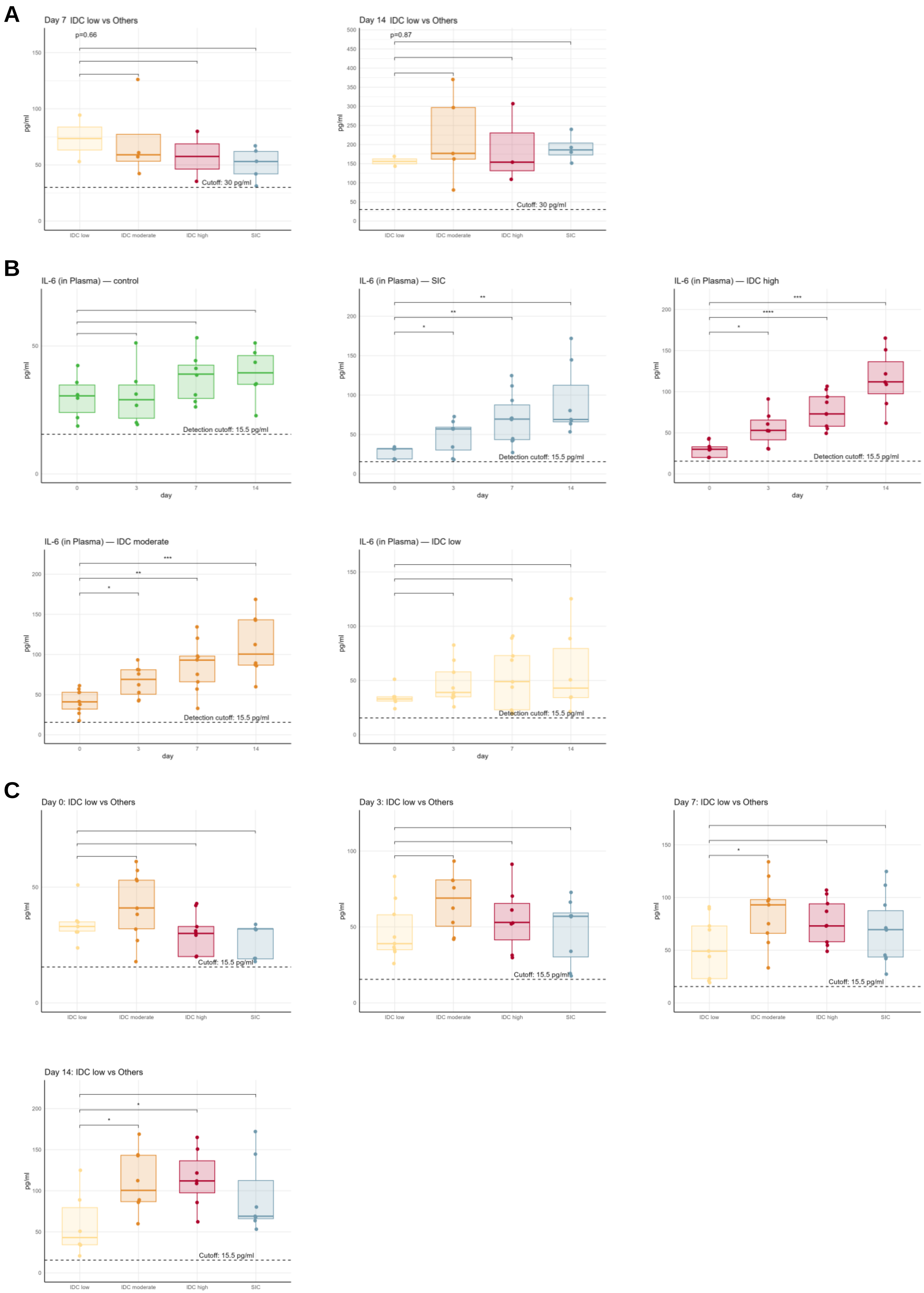

Appendix
Figure 5

A lung-IDC dpi3

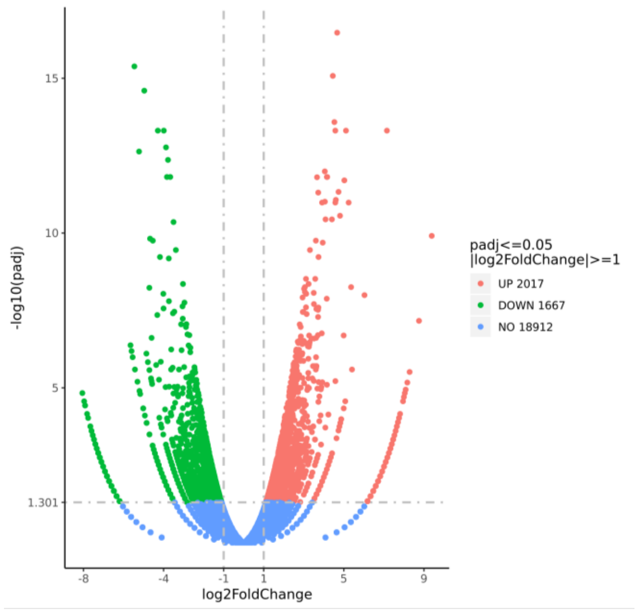

B lung-SIC dpi3

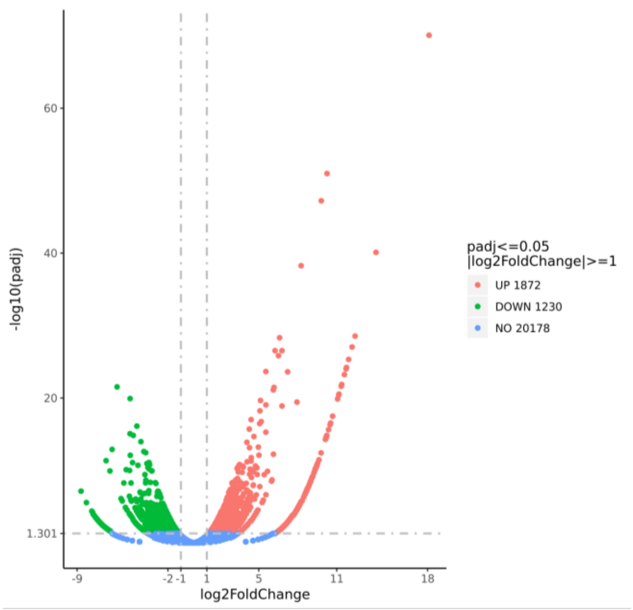

C PBMCs-IDC dpi3

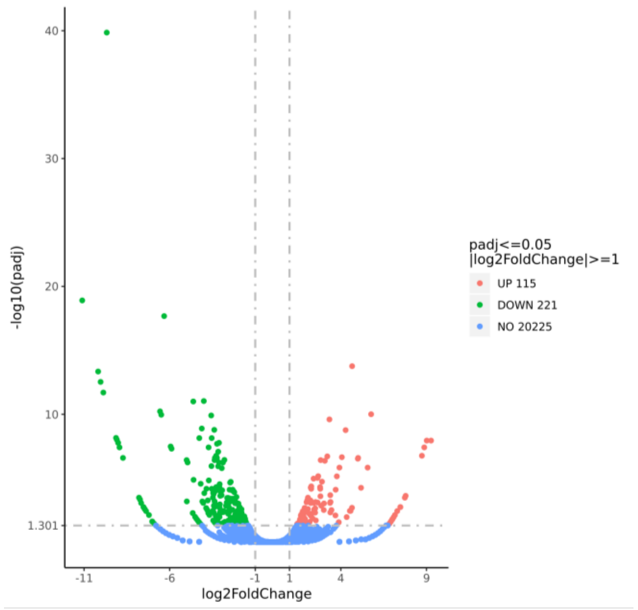

D PBMCs-SIC dpi3

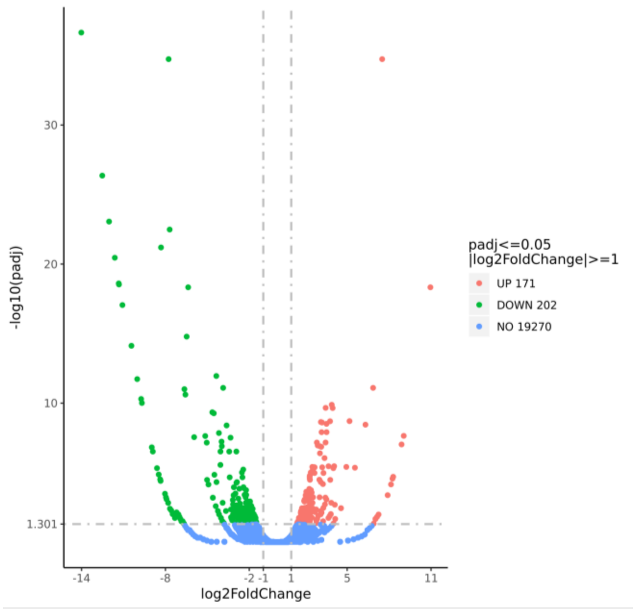

Appendix
Figure 6

A lung-IDC dpi7

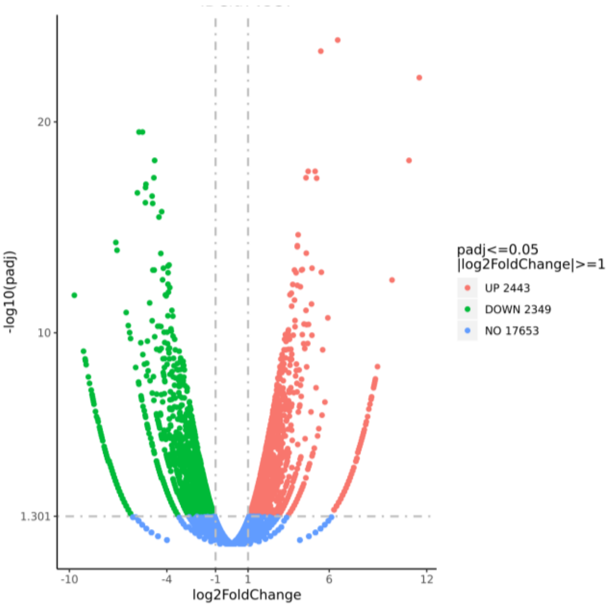

B lung-SIC dpi7

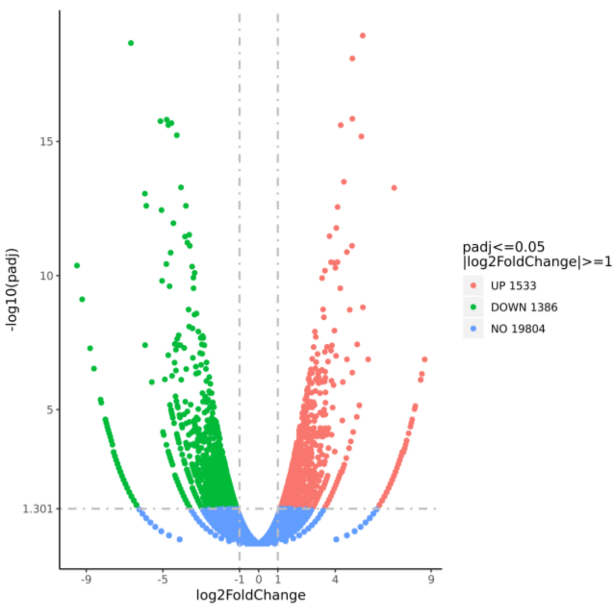

C PBMCs-IDC dpi7

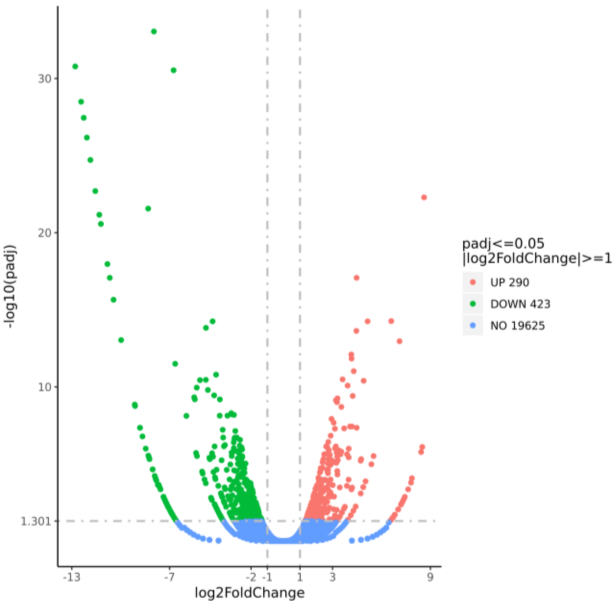

D PBMCs-SIC dpi7

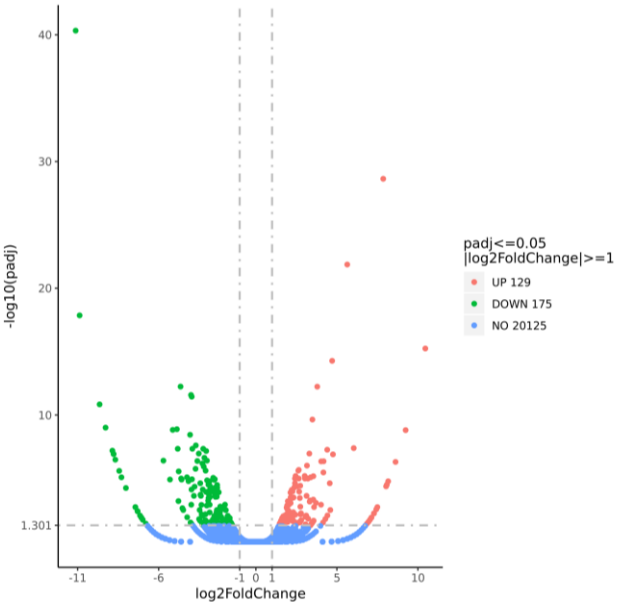

Supplement: Appendix Figure.pdf [file TEMI_A_2511134_SM1184.pdf]
